# Supplementary material for: Conditional chemoconnectomics (cCCTomics) as a strategy for efficient and conditional targeting of chemical transmission
Source: eLife. 2024 Apr 30;12:RP91927. doi: 10.7554/eLife.91927 (PMC11060718; doi:10.7554/eLife.91927)
Supplement: Figure 3—source data 3. [file elife-91927-fig3-data3.zip › Figure 3 source data3 part 1.zip!/Figure 3-Source data 3-part1/1082-9-CRISPResso_Report_zod2ewbs.zip!/Nucleotide_percentage_quilt.pdf]

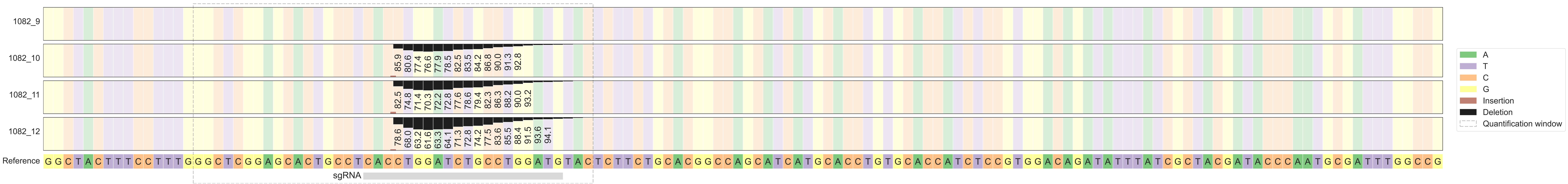
1082_9
1082_10
85.9 80.6 77.4 76.6 77.9 78.5 82.5 83.5 84.2 86.8 90.0 91.3 92.8
1082_11
82.5 74.8 71.4 70.3 72.2 72.8 77.6 78.6 79.4 82.3 86.3 88.2 90.0 93.2
1082_12
78.6 68.0 63.2 61.6 63.3 64.1 71.3 72.8 74.2 77.5 83.6 85.5 88.4 91.5 93.6 94.1
Reference
GGCTACTTTCCTTTGGGCTCGGAGCACTGCCTCACCTGGATCTGCCTGGATGTACTCTTCTGCACGGCCAGCATCATGCACCTGTGCACCATCTCCGTGGACAGATATTTATCGCTACGATACCCAATGCGATTTGGCCG
sgRNA
A
T
C
G
Insertion
Deletion
Quantification window
